# Hollow ZIF-8 Nanoworms from Block Copolymer Templates

**DOI:** 10.1038/srep15275

**Published:** 2015-10-16

**Authors:** Haizhou Yu, Xiaoyan Qiu, Pradeep Neelakanda, Lin Deng, Niveen M. Khashab, Suzana P. Nunes, Klaus-Viktor Peinemann

**Affiliations:** 1 Advanced Membranes and Porous Materials Center, 4700 King Abdullah University of Science and Technology (KAUST), Thuwal 23955-6900, Kingdom of Saudi Arabia; 2 Biological and Environmental Science and Engineering Division, 4700 King Abdullah University of Science and Technology (KAUST), Thuwal 23955-6900, Kingdom of Saudi Arabia

## Abstract

Recently two quite different types of “nano-containers” have been recognized as attractive potential drug carriers; these are wormlike filamenteous micelles (“filomicelles”) on the one hand and metal organic frameworks on the other hand. In this work we combine these two concepts. We report for the first time the manufacturing of metal organic framework nanotubes with a hollow core. These worm-like tubes are about 200 nm thick and several μm long. The preparation is simple: we first produce long and flexible filament-shaped micelles by block copolymer self-assembly. These filomicelles serve as templates to grow a very thin layer of interconnected ZIF-8 crystals on their surface. Finally the block copolymer is removed by solvent extraction and the hollow ZIF-8 nanotubes remain. These ZIF-NTs are surprisingly stable and withstand purification by centrifugation. The synthesis method is straightforward and can easily be applied for other metal organic framework materials. The ZIF-8 NTs exhibit high loading capacity for the model anti cancer drug doxorubicin (DOX) with a pH-triggered release. Hence, a prolonged circulation in the blood stream and a targeted drug release behavior can be expected.

The need for drug nanocarriers that efficiently target diseased areas in the body arises because drug efficacy is often altered by nonspecific biodistribution in cells and tissue and because some drugs are rapidly metabolized in or excreted from the body^1^,^2^. Currently, many materials are being used as nanocarriers, including liposomes^3^, polymer nanoparticles^4^, micelles^5^, dendrimers^6^, and inorganic nanoparticles made of iron oxide, quantum dots, gold or metal organic frameworks (MOFs)^7^,^8^,^9^,^10^. More recently, the shape of nano/micro particles has been recognized as a critical determinant in regulating vascular transport^11^, cellular uptake^12^ and differential organ accumulation^13^. For example, discoidal particles have demonstrated advantages in navigating and targeting diseased vasculature, as compared with traditional spherical particles^14^. An important study has illustrated the dramatic role that shape can play in the function of engineered nanoparticles by synthesizing ‘filomicelles’, worm-like filamentous micelles, and by taking advantage of the morphological features of viruses^15^. These virus-mimicking filomicelles, composed of self-assembled block copolymers, effectively evade the reticuloendothelial system (RES), resulting in remarkably long blood circulation times (approximately one week). This is significantly longer than the blood circulation time of stealth liposomes^16^. The result is remarkable, given that micrometer-sized rigid spheroids are cleared from the circulation almost immediately^17^. In addition, when loaded with the widely prescribed anti-cancer drug paclitaxel, filomicelles also shrank tumors more effectively than did either the free drug or spherical nanocarriers^16^. The *in vivo* effectiveness of such carriers with nonspherical shapes is likely to be studied in more depth in the coming years^18^.

Recently, MOFs have been recognized as attractive potential drug carriers^19^,^20^. Here, we report for the first time a simple approach to producing hollow nanoworms that are formed by inter-grown metal-organic framework nano-crystals. We use long and flexible filament-shaped micelles of a self-assembled amphiphilic block copolymer (BCP) in selective solvent mixtures as templates. Via a two-cycle growth process, a thin layer of zeolite imidazolate frameworks-8 (ZIF-8) could be easily fabricated on the surface of these BCP micelles. We further immersed the hybrid BCP@ZIF-8 structures in a common solvent for the copolymer blocks to completely remove the organic polymer portion, yielding filamentous ZIF-8 nanotubes (NTs) that exhibit high loading capacity and pH-responsiveness. We envision that when carefully designed, the worm-like ZIF-8 NTs might mimic both the structure and function of sophisticated biological systems (*i.e.,* filament-shaped viruses) and would thus offer selective targeting while evading the immune system during prolonged circulation.

## Results and Discussion

**Figure 1:**
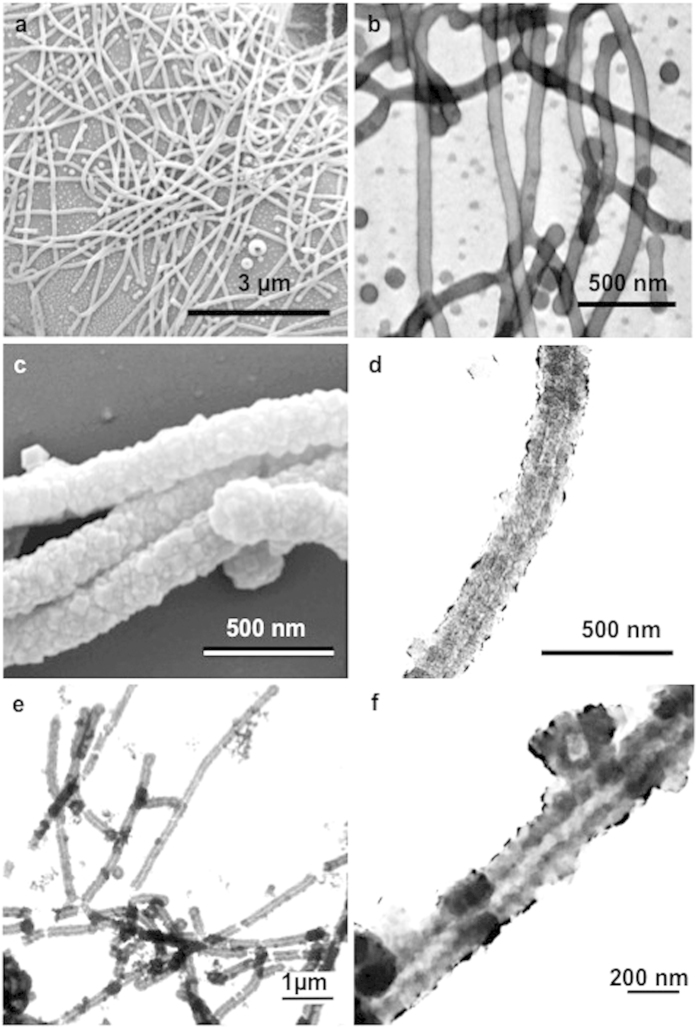
(**a**) SEM and (**b**) I_2_-stained TEM images of the BCP filament-shaped micelles; (**c**) SEM and (**d**) TEM images of the hybrid BCP@ZIF-8 structures; (**e**,**f**) TEM images of the final filamentous ZIF-8 NTs at different magnifications after BCP removal.

Our starting material was a diblock copolymer with a hydrophilic chain of poly-4-vinylpyridine (P4VP) and a hydrophobic chain of polystyrene (PS). Toluene and methanol were chosen as selective solvents with preferential interactions with PS and P4VP, respectively. Factors such as the composition and concentration of the copolymer, the nature and content of the solvents, and the corresponding block-block and block-solvent *Flory–Huggins* parameters, χ^21^, significantly affect the morphology of the copolymer assemblies in solution. χ can be roughly estimated by analyzing the difference between the solubility parameters (*δ*) of each component. When the values of the solubility parameters for the polymer and solvent are similar, dissolution of the polymer is optimal. In block copolymers, the differences in the solubility of each block and solvent determine which block is preferentially exposed to the solvent. Table S1 lists the dispersive (*δ*^*d*^), polar (*δ*^*p*^) and H-bond (*δ*^*H*^) contributions from the single solvents, solvent mixtures and copolymer blocks investigated here. (*δ*_*solvent*_ − *δ*_*block*_)^2^ gives a good indication of how strong the interaction between the blocks and the solvent is, with larger values indicating poorer interactions^22^. We chose a 3:7 (v/v) mixture of toluene and methanol as the solvent mixture. Table S1 shows that the differences in the solubility parameters are much larger between styrene blocks and the solvent mixture ((*δ*^*P*^_*solvent mix*_ − *δ*_S_)^2^ = 60.8, (*δ*^*H*^_*solvent mix*_ − *δ*_*S*_)^2^ = 137) than they are between 4-vinylpyridine and the solvent mixture ((*δ*^*P*^_*solvent mix*_ − *δ*_*4VP*_)^2^ = 2.6, (*δ*^*H*^_*solvent mix*_ − *δ*_*4VP*_)^2^ = 81). The P4VP blocks tend to be exposed to the selective solvent mixture, as confirmed by the TEM image shown in Fig. 1b. The P4VP domain (periphery of the micelles) became dark after being selectively stained by I_2_.

In our study, the filament-shaped micelles were formed by adding 10 mg of PS-*b*-P4VP directly to a mixture of toluene (0.3 ml) and methanol (0.7 ml) under stirring. The resultant worm-like micelles had lengths varying from 5 to 10 μm (Fig. 1a and Fig. S2). Geng *et al.*^15^ claimed that ~8 μm is an optimal length for filomicelles used as drug carriers injected in rodents. This length is close to the diameter of circulating red blood cells (RBCs) in rodents. They showed that longer filomicelles rapidly fragmented to this size, whereas shorter filomicelles were quickly eliminated.

Template-directed synthesis is generally accepted as a simple, high-throughput, and cost-effective procedure that allows the straightforward production of hybrid materials, often in only one step^23^,^24^. In our study, the lone electron pair on nitrogen of P4VP acted as a strong ligand for coordination with Zn^2+^ ions. After adding the as-synthesized PS-*b*-P4VP filament-shaped micelles to a precursor methanol solution containing Zn(NO_3_)_2_ and 2-methylimidazole (HMeIM) at 70 °C for 10 min, a seed layer of ZIF-8 was grown on the surface of the micelles (Figure S1). To grow a compact and gap-free (highly adhesive) ZIF-8 layer on the surface, two cycles of the solvothermal ZIF-8 growth reaction were performed. ZIFs, as a new subfamily of MOFs, have been attracting particular attention as drug delivery carriers in view of their biocompatibility and their ability to load large amounts of drugs^25^. In particular, the ZIF-8 synthesized by Yaghi and co-workers in 2006 resembled a regular zeolitic sodalite (sod) topology^26^. The intriguing structural features of large pores and large surface areas^27^,^28^ make ZIF-8 the best established ZIF material with a variety of impressive applications^29^.

**Figure 2:**
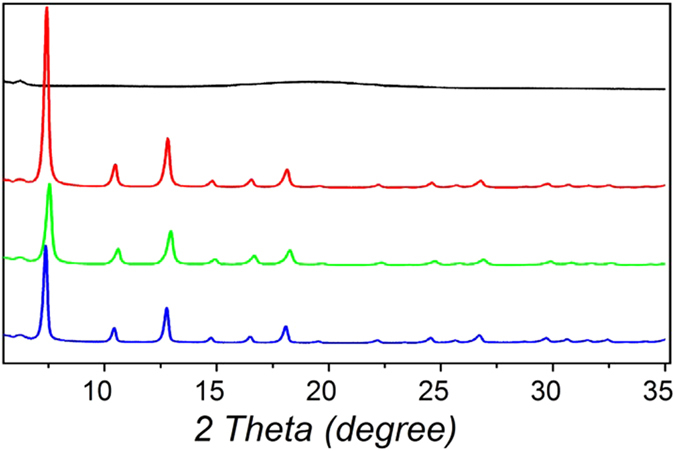
X-ray diffraction (XRD) patterns of BCP filament-shaped micelles (black), hybrid BCP@ZIF-8 structures (red), filamentous ZIF-8 NTs (green) and ZIF-8 NPs (blue).

Some recent studies have concentrated on the generation of porous membranes, thin-films, micrometer-sized particles or hollow spherical forms from ZIF-8^30^,^31^,^32^,^33^,^34^,^35^,^36^,^37^. However, the fabrication of one-dimensional (1D) ZIF-8 nanostructures, such as nanotubes, has not been studied. Here, the desired hybrid BCP@ZIF-8 structures were easily separated from the unwanted ZIF-8 nano-crystals (Figure S3a) via centrifugation. The hybrid BCP@ZIF-8 structures were characterized by scanning electron microscopy (SEM) and transmission electron microscopy (TEM). The diameter changed from 80 nm for the initial BCP micelles to *ca.* 220 nm after two cycles of the solvothermal ZIF-8 growth reaction, indicating the generation of ZIF-8 shells with thicknesses of *ca.* 70 nm surrounding the BCP micelles (Fig. 1d). SEM (Fig. 1c) and TEM (Fig. 1d) images indicate the formation of ZIF-8 shells consisting of numerous nano-sized ZIF-8 crystals. To verify the successful growth of ZIF-8, we utilized X-ray diffraction (XRD), Fourier transform infrared (FT-IR) measurements and energy dispersive X-ray spectrometry (EDX). In the XRD pattern, the diffraction peaks^38^ at *ca.* 7.3° and 12.7° confirm the presence of the ZIF-8 layer (Fig. 2). Figure S4 shows the FT-IR spectra of BCP micelles and hybrid BCP@ZIF-8 structures. The peak at 3100–3000 cm^−1^ can be assigned to the aromatic rings^39^, indicating the presence of polystyrene blocks. The bands at 3135 and 2929 cm^−1^ are attributed to the aromatic and the aliphatic C–H stretch of the imidazole^40^, respectively, indicating the presence of ZIF-8 on the surface of the BCP micelles. The observation of zinc atoms in the EDX spectrographs supported the possibility that new materials containing zinc atoms had been generated on the surfaces of the BCP micelles (Figure S5).

Immersing the as-prepared hybrid BCP@ZIF-8 structures in *N,N'*-dimethylformamide (DMF), which is a common solvent for both PS and P4VP chains, led to the complete removal of BCP and produced the filamentous ZIF-8 nanotubes. The tubular shape was properly maintained after removing the BCP core (Fig. 1e,f). The ZIF-8 structure was confirmed by XRD (Fig. 2). An XRD pattern typical of the ZIF-8 sodalite structure was confirmed for the nanotubes (NTs), before and after removal of the block copolymer, as evidenced by the good agreement with the XRD patterns obtained for pure ZIF-8 spherical nanoparticles (NPs) directly formed without templates and with previously reported XRD analysis of ZIF-8^26^. In addition, the disappearance of the peak at 3100–3000 cm^−1^ from the FT-IR spectra (Figure S3) indicates that BCP was completely removed. Moreover, the chemical composition of the filamentous ZIF-8 NTs was characterized by the EDX spectrum, revealing a typical pure ZIF-8 composition. In the EDX spectra (Figure S5), the detection of zinc atoms after the creation of the ZIF-8 shell portion and the decrease in carbon atoms as a result of the elimination of the PS-*b*-P4VP domains are in good agreement with the compositional changes occurring during the formation processes of filamentous ZIF-8 NTs. This general synthetic method described here could be extended to the synthesis of other ZIFs, *i.e.,* ZIF-7 NTs (Figure S7).

**Figure 3:**
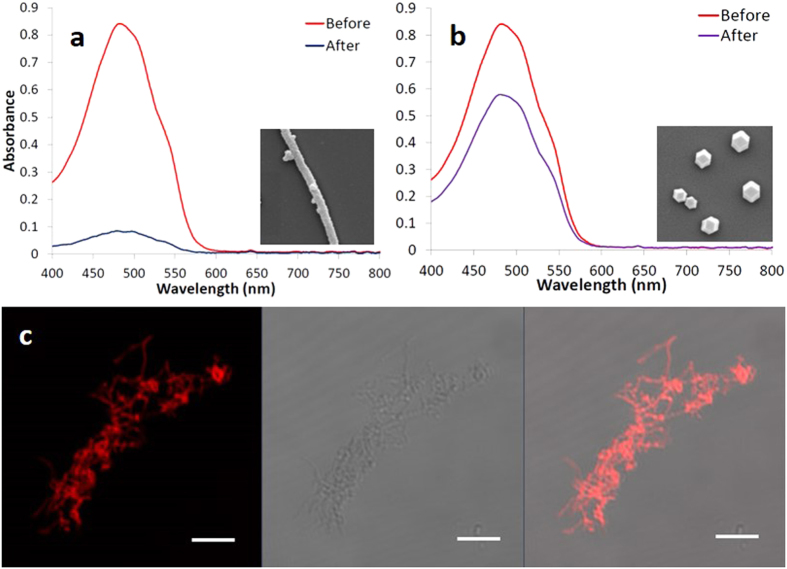
UV-Vis absorption spectra of DOX solutions before and after interactions with (a) hollow filamentous ZIF-8 NTs and (b) compact ZIF-8 NPs. The insets are the SEM images of the ZIF-8 NTs and NPs, respectively; (**c**) shows the LSCM images of filamentous ZIF-8 NTs loaded with DOX. The image on the left is the fluorescence image; the image in the middle is the transmission image; the image on the right is the overlay of these two images. The scale bars correspond to 5 μm.

One of the most active research areas for drug delivery systems currently is establishing drug release in an acidic environment^41^,^42^. The extracellular pH values of tumors, especially inside their endosomal (pH = 5.5–6.0) and lysosomal (pH = 4.5–5.0) compartments, is lower than the pH in normal tissues and in the bloodstream^43^,^44^. Using a pH-responsive drug vehicle might reduce the release of the drug during its transportation in the blood circulation system and improve the effective release of the anti-tumor drug inside tumor cells. Because ZIF-8 crystals are porous, the hollow cores of our ZIF-8 NTs became accessible through the nanopores in the shells. Due to the large hollow core volume of the ZIF-8 NTs, high drug loading capacity is expected. In addition, when suspended in acid solution, the ZIF-8 NTs began to decompose quickly. To evaluate the loading capacity of the ZIF-8 NTs, we used doxorubicin (DOX), an anti-cancer drug, as a model drug. Figure 3a shows the UV-Vis absorption spectra of DOX before and after interaction with the (hollow) filamentous ZIF-8 NTs. The absorption intensity of supernatant DOX decreased dramatically after the interaction with ZIF-8 NTs, indicating the significant decrease of the drug concentration in solution and verifying the effective storage of DOX in the hollow, elongated ZIF-8 NTs. The high loading capacity of DOX into the ZIF-8 NTs could reach 350%, *i.e.*, 1 mg filamentous ZIF-8 NTs could load about 3.5 mg DOX, which is almost three times more than that loaded into ZIF-8 non-hollow particles synthesized without block copolymer templates (Figure S3b), *i.e.,* 1 mg of the non-filamentous ZIF-8 particles could load 1.2 mg DOX (Fig. 3b). These findings are useful because only small amounts of carrier material would be required for the administration of high dosages in clinical applications. The strong red fluorescent signal generated from DOX in the laser scanning confocal microscopy (LSCM) images (Fig. 3c) also showed that DOX efficiently loaded into the ZIF-8 NTs.

**Figure 4:**
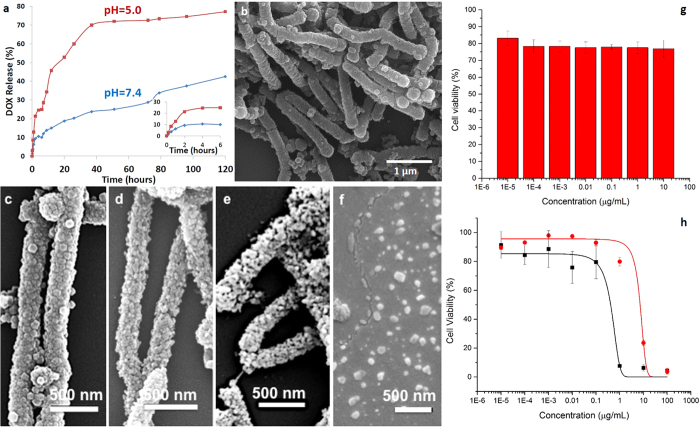
(**a**) DOX release from the filamentous ZIF-8 NTs. The inset shows the expanded plot for the release process from 0 to 6 h; SEM images of the filamentous ZIF-8 NTs immersed in (**b**) phosphate-buffered saline (pH = 7.4) for one week and acidic phosphate buffer (pH = 5.0) for (**c**) 0 min, (**d**) 8 min, (**e**) 1 h and (**f**) one week; (**g**) Cytotoxicity of ZIF-8 NTs; (**h**) Cytotoxicity of doxorubicin (black) and doxorubicin-loaded ZIF-8 NTs (red).

Besides drug loading, time-dependent release is the other important parameter for drug efficacy and delivery in a controlled manner within a therapeutic range. The *in vitro* release rate of DOX from the ZIF-8 NTs could be controlled by changing pH values at 37 °C. At the physiological pH of 7.4 in phosphate-buffered saline, DOX was released in a very slow fashion and the cumulative release of DOX was only about 25% even after 50 h. In contrast, the nanotubes exhibited a much faster drug release rate at pH 5.0, under which conditions more than 72% of DOX was released after 50 h (Fig. 4a). We attributed this difference in release time to the pH-sensitivity of the filamentous ZIF-8 NTs. After immersion in phosphate-buffered saline, the samples were periodically observed under SEM and found to be unchanged. The tubular shape and the surface were well maintained even after one week at pH 7.4 (Fig. 4b). The hydrolysis of Zn-imidazole MOFs might depend on the anionic components in solution. Therefore a simulated body fluid with inorganic components similar to those of human blood plasma (Table S2) was prepared to check the stability of the ZIF-NTs in this environment (details in supplementary information). Again, no change in shape could be observed. In contrast, after immersion in acidic phosphate buffer at pH 5.0, the surface of the ZIF-8 NTs lost its shape and surface continuity after 8 minutes and they were further decomposed after one hour (Fig. 4c–e and Figure S6), resulting in the fast release of DOX. After one week, the ZIF-8 NTs were thoroughly disassembled to small ZIF-8 particles (Fig. 4f). These results suggest that the ZIF-8 NTs decompose at a pH of 5, a feature that can be exploited during drug delivery, especially in targeting cancerous tissues.

The cytotoxicity of ZIF-8 NTs was evaluated by incubating HeLa cells with different concentrations of ZIF-8 NTs (1 × 10^−5^ to 10 μg/mL) for 24 h followed by standard Cell Counting Kit-8 (CCK-8) assay (Fig. 4g). According to a previous study^25^ ZIF-8 nanoparticles did not show obvious toxicity. Herein, the moderate toxicity of ZIF-8 NTs could be ascribed to their micro-sized length which could disturb and destroy the cell membrane during cell internalization and induce the cell death^45^. However, the toxicity could be reduced by decreasing the dosage thanks to the high loading capacity of the ZIF-8 NTs in this work. The cytotoxicity of free DOX and DOX-loaded ZIF-8 NTs were also evaluated by incubating HeLa cells with different concentrations of DOX (1 × 10^−5^ to 100 μg/mL) for 24 h followed by standard CCK-8 assay (Fig. 4h). DOX-loaded ZIF-8 NTs showed relatively lower toxicity (red) than free DOX (black) which matches well with the cumulative release of DOX from ZIF-8 NTs in Fig. 4a.

**Figure 5:**
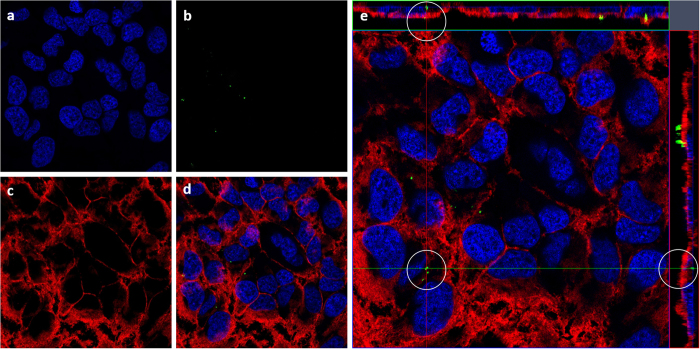
CLSM images of HeLa cells after incubation with ZIF-8 NTs.

The cellular internalization of ZIF-8 NTs is investigated by incubating ZIF-8 NTs with HeLa cells for 12 h at 37 °C. The co-localization of ZIF-8 NTs was then determined using confocal laser scanning microscopy (CLSM). The nuclei (Fig. 5a) and cell membrane (Fig. 5c) were stained by Hoechst 33342 and CellMask^TM^ deep red separately, while ZIF-8 NTs was labelled with bright green FITC (Fig. 5b). Merged image is shown in Fig. 5d. Z-stack (Fig. 5e) was performed to identify the ZIF-8 NTs intracellular location. The internalized ZIF-8 NTs (white circles) suggest that ZIF-8 NTs were uptaken by the cells (Fig. 5e).

In summary, we developed a simple strategy to fabricate worm-like ZIF-8 NTs by using self-assembled BCP filomicelles as templates for the growth of connected ZIF-8 nanocrystals. The resulting filamentous ZIF-8 NTs exhibit high loading capacity for DOX and a pH-sensitive release property. *in vitro* studies indicated that the cytotoxicity of the ZIF-8 NTs is moderate and that the ZIF-8 NTs could be taken up by HeLa cells. The general synthetic method described here might be extended to the synthesis of other functional nanomaterials for various biomedical potential applications.

## Methods

### Materials

Polystyrene-*b*-poly-4-vinylpyridine (PS-*b*-P4VP, 188,000-*b*-64,000 g/mol) was obtained from Polymer Source, Inc. (Canada). Toluene and methanol were supplied by Fluka Analytical. Doxorubicin hydrochloride (DOX, 580.00 g/mol), Zinc nitrate hexahydrate (Zn(NO_3_)_2_·6H_2_O), 2-methylimidazole, benzimidazole, fluorescein isothiocyanate (FITC) and Cell Counting Kit-8 (CCK-8) were purchased from Sigma-Aldrich (USA). All chemicals were used as purchased directly without further purification. The human cervical tumor cell line (HeLa) was obtained from ATCC (USA). Eagle’s minimal essential medium (EMEM), fetal bovine serum (FBS), Dulbecco's phosphate-buffered saline (DPBS), penicillin-streptomycin, Hoechst 33342 and CellMask™ deep red plasma membrane stain were purchased from Life technologies (USA).

### Micelle preparation

To prepare the BCP filament-shaped micelles, 10 mg of BCP was directly dissolved in the mixtures of toluene (0.3 ml) and methanol (0.7 ml) with stirring at room temperature. After 10 hours the mixtures were diluted to the concentration of 1 mg/ml with 10 times of methanol. The final BCP filament-shaped micelles solution was ready for use.

### Preparation of BCP@ZIF-8 structures

A equivalent volume mixture (4 ml) of 2-methylimidazole (HMeIM, 20 mg/ml) and Zn(NO_3_)_2_·6 H_2_O (7.5 mg/ml) was prepared in methanol as a precursor solution. Then BCP filament-shaped micelles solution (1 mg/ml) with the same volume (4 ml) was added to the precursor solution. The resulting mixture was placed in an oil bath (70 °C) for 10 min. Hybrid BCP@ZIF-8 structures generated during this time were isolated by cooling the reaction mixture to room temperature, collecting the precipitate by centrifugation, and washing the precipitate several times with methanol. During centrifugation process, the desired hybrid BCP@ZIF-8 structures were easily separated from the unwanted ZIF-8 nano-crystals due to their density difference. The second ZIF-8 growth cycle was continuously conducted to achieve good growth of a compact and gap-free ZIF-8 layer using a fresh precursor solution.

### Preparation ZIF-8 nanotubes

The as-prepared hybrid BCP@ZIF-8 structures were immersed in *N,N’*-dimethylformamide (DMF) to dissolve the BCP portions yielding the filamentous ZIF-8 NTs. The resulting products were collected by centrifugation and were then washed several times with DMF and methanol.

### Preparation ZIF-8 nanoparticles

A precursor solution was prepared by mixing equivalent volume of 2-methylimidazole (HMeIM, 20 mg/ml) and Zn(NO_3_)_2_·6H_2_O (7.5 mg/ml) was prepared in methanol. The mixture was placed in an oil bath (70 °C) for 10 min. The products were collected by centrifugation and were then washed several times with methanol.

### Characterization

Fourier transform infrared (FT-IR) spectra were obtained on a Nicolet iS 10 FT-IR spectrometer (Thermo Scientific). Transmission electron microscopy (TEM) images were obtained using a Tecnai 12 (FEI Company) operating at 120 keV. Scanning electron microscopy (SEM) images and energy dispersive X-ray spectrometry (EDX) were obtained using environmental FESEM (FEI Quanta 600 series). Imaging was carried out at 5 kV with a working distance of 10 mm. X-ray diffraction (XRD) patterns were carried out with a diffractometer Bruker B8 Advance, Cu radiation (40 kV, 40 mA scan range 5 to 35 degree).

### Drug loading

1 mg of filamentous ZIF-8 NTs or ZIF-8 NPs was dispersed in 1 ml of DOX solution with the DOX concentration of 5 mg/ml. The mixture was centrifuged to collect the DOX-loaded ZIF-8 NTs or NPs after stirring for 3 days at room temperature. To calculate the amount of loaded DOX, the contents of original DOX and the supernatant were determined by NanoDrop 2000/2000c spectrophotometer (Thermo Fisher Scientific) at 480 nm. The DOX loading efficiency (LE) can be calculated by the following formula:

*LE* (*%*) = [*m*(*original DOX*) *−* *m*(*DOX in supernatant*)]*/m*(*original DOX*) × *100%*

### Drug-release studies

A commercial solution of phosphate-buffered saline (PBS) at the physiological pH of 7.4 was used after dilution in water (10 times). Acidic phosphate buffer solutions (pH = 5.0) are adjusted by mixing potassium dihydrogen phosphate (KH_2_PO_4_) and disodium hydrogen phosphate (Na_2_HPO_4_) (both from Fluka) in Milli-Q water and are used within three days of preparation. 1 mg ZIF-8 NTs was immersed in 1 ml DOX solution (0.5 mg/ml) for 2 hours. Nanodrop™ 2000/2000c result indicated that the concentration of the supernatant was 0.15 mg/ml. That means 0.35 mg was already loaded into the filamentous ZIF-8 NTs. DOX loaded ZIF-8 NTs were immersed in PBS (pH = 7.4) and acidic phosphate buffer solutions (pH = 5.0) at 37 °C, respectively. The released DOX was sampled at defined time periods and their concentrations were measured using a NanoDrop™ 2000/2000c Spectrophotometer.

### Laser scanning confocal microscopy (LSCM)

The DOX loaded ZIF 8 NTs were imaged using a confocal microscope (Zeiss LSM 710) equipped with a 60× or 100× oil immersion objective. ZEN 2009 software (Carl Zeiss Microscopy, GmbH) was used to collect and orient the imaging data.

### Cells viability of HeLa cells treated with ZIF-8 NTs

The cytotoxicity of ZIF-8 NTs incubated with HeLa cells were evaluated using the CCK-8 assay. Cells were seeded at a density of 5 × 10^3^ cells per well in 96-well flat bottom plates and incubated with EMEM medium containing 10% FBS and 0.1% penicillin-streptomycin at 37 °C in a humidified 5% CO_2_ atmosphere for 12 h. After cell attachment, they were washed with DPBS and incubated with different concentrations (10, 1, 0.1, 0.01, 1 × 10^−3^, 1 × 10^−4^ and 1 × 10^−5^ μg/mL) of ZIF-8 NTs solutions in EMEM media for 24 h. Cell viability was evaluated by the CCK-8 colorimetric procedure.

### Intracellular localization and internalization of FTIC-ZIF-8 NTs

HeLa cells were seeded on glass cover slides, and cultured in EMEM medium containing 10% FBS and 0.1% penicillin-streptomycin at 37 °C in a humidified 5% CO_2_ atmosphere. After cell attachment, the medium was replaced by fresh medium containing 20 μg/mL of FTIC-ZIF-8 NTs, followed by incubation for 12 h. Cells on cover slides were washed twice with DPBS, then fixed with 4% paraformaldehyde for 1 h and washed 3 times with DPBS. Nuclei were then stained with Hoechst 33342 for 20 min and washed 3 times with DPBS. Cell membrane was stained with CellMask^TM^ deep red plasma membrane dye for 2 min and washed for 3 times with DPBS. Finally, cells were observed with confocal laser scanning microscopy (CLSM, Zeiss LSM 710 inverted confocal microscope).

## Additional Information

**How to cite this article**: Yu, H. *et al.* Hollow ZIF-8 Nanoworms from Block Copolymer Templates. *Sci. Rep.* **5**, 15275; doi: 10.1038/srep15275 (2015).

## Supplementary Material

Supplementary Information
