# Supplementary material for: Hollow ZIF-8 Nanoworms from Block Copolymer Templates
Source: Sci Rep. 2015 Oct 16;5:15275. doi: 10.1038/srep15275 (PMC4607995; doi:10.1038/srep15275)
Supplement: Supplementary Information [file srep15275-s1.doc]

Hollow ZIF-8 Nanoworms from Block Copolymer Templates

Haizhou Yu, Xiaoyan Qiu, Pradeep Neelakanda, Lin Deng, Niveen M. Khashab, Suzana P. Nunes and Klaus-Viktor Peinemann

**Supporting Information:**

**Preparation ZIF-8 nanoparticles.** A precursor solution was prepared by mixing equivalent volume of 2-methylimidazole (HMeIM, 20 mg/ml) and Zn(NO_3_)_2_·6H_2_O (7.5 mg/ml) was prepared in methanol. The mixture was placed in an oil bath (70 °C) for 10 min. The products were collected by centrifugation and were then washed several times with methanol.

**Preparation ZIF-7 nanotubes.** A equivalent volume mixture (4 ml) of benzimidazolate (PhIm, 15 mg/ml) and Zn(NO_3_)_2_·6H_2_O (6 mg/ml) was prepared in methanol as a precursor solution. Then BCP filament-shaped micelles solution (1mg/ml) with the same volume (4 ml) was added to the precursor solution. The resulting mixture was placed in an oil bath (70 °C) for 15 min. Hybrid BCP@ZIF-7 structures generated during this time were isolated by cooling the reaction mixture to room temperature, collecting the precipitate by centrifugation, and washing the precipitate several times with methanol. During centrifugation process, the desired hybrid BCP@ZIF-7 structures were easily separated from the unwanted ZIF-7 nano-crystals due to their density difference. The second ZIF-7 growth cycle was continuously conducted to achieve good growth of a compact and gap-free ZIF-7 layer using a fresh precursor solution. The as-prepared hybrid BCP@ZIF-7 structures were immersed in *N,N’*-dimethylformamide (DMF) to dissolve the BCP portions yielding the filamentous ZIF-7 NTs. The resulting products were collected by centrifugation and were then washed several times with DMF and methanol.

**Table S1.** Hansen solubility^a^ contributions for solvents, solvent mixtures and copolymer blocks.

|  | δ_contributions_ | | |
| --- | --- | --- | --- |
|  | d | p | h |
| δ_Toluene_ | 18.0 | 1.4 | 2.0 |
| δ_Methanol_ | 15.1 | 12.3 | 22.3 |
| δ_Styrene_ | 18.6 | 1.0 | 4.1 |
| δ_4-Vinyl Pyridine_ | 18.1 | 7.2 | 6.8 |
| δSolvent mixture  (32 wt% toluene/68 wt% methanol) | 16.0 | 8.8 | 15.8 |
| (δ_T_- δ_4VP_)^2^ | 0.01 | 33.6 | 23.0 |
| (δ_T_- δ_S_)^2^ | 0.4 | 0.2 | 4.4 |
| (δ_M_- δ_4VP_)^2^ | 9 | 26 | 240 |
| (δ_M_- δ_S_)^2^ | 12 | 128 | 331 |
| (δ_Solvent mix_- δ_S_)^2^ | 6.8 | 60.8 | 137 |
| (δ_Solvent mix_- δ_4VP_)^2^ | 4.4 | 2.6 | 81 |

^a^ C. M. Hansen, Hansen solubility parameters: a user’s handbook, CRC Press, 1999.

**Table S2.** Composition of simulated body fluid

| **Reagent** | **Amount in grams** |
| --- | --- |
| NaCl | 7.90 g |
| NaHCO_3_ | 0.35 g |
| KCl | 0.22 g |
| K_2_HPO_4_・3H_2_O | 0.22 g |
| MgCl_2_・6H_2_O | 0.30 g |
| CaCl_2_ | 0.27 g |
| Na_2_SO_4_ | 0.07 g |
| 1 M HCL | 40 ml |
| Tris buffer | 6 g |
| water | 945g |

Amount of HCl and tris buffer mentioned in the table are approximate values because they were added according to the pH 7.4 adjustment. In the reference link below they have used (CH_2_OH)_3_CNH_2_ as base buffer instead Tris buffer.

Reference: <http://mswebs.naist.jp/LABs/tanihara/ohtsuki/SBF/>

**
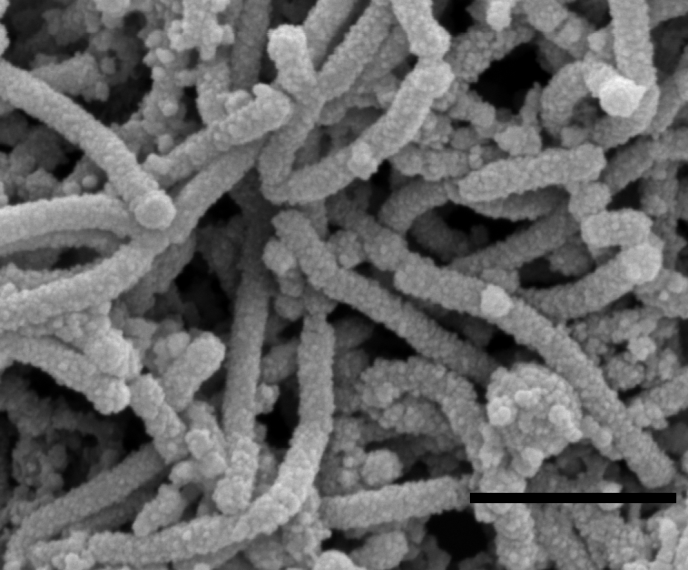

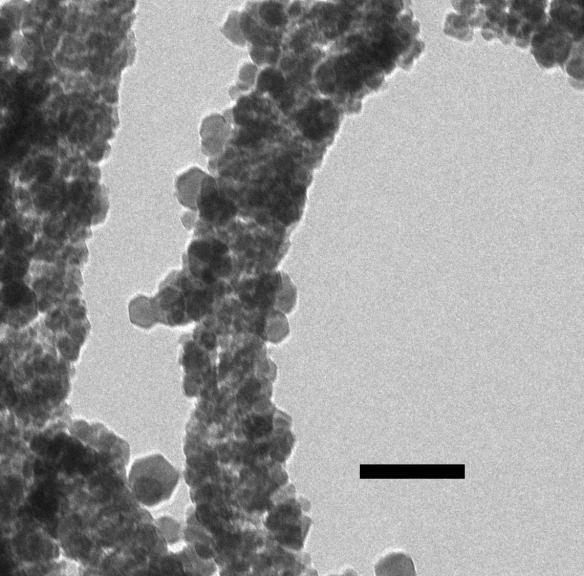
**

**200 nm**

**1 µm**

**(a)**

**(b)**

**Figure S1.** SEM (a) and TEM (b) images of the BCP filament-shaped micelles after the seeding step (one cycle) with ZIF-8.


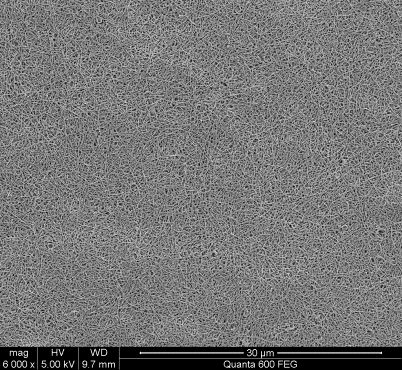

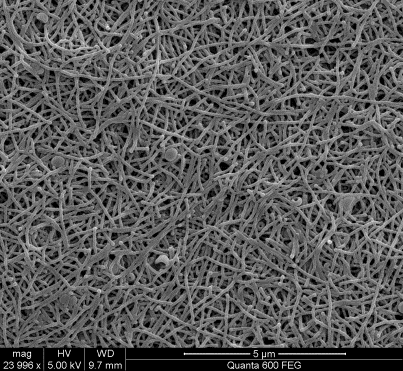

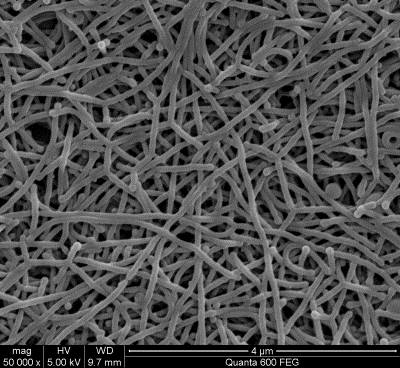


**Figure S2.** Wormlike micelles at different magnifications

**
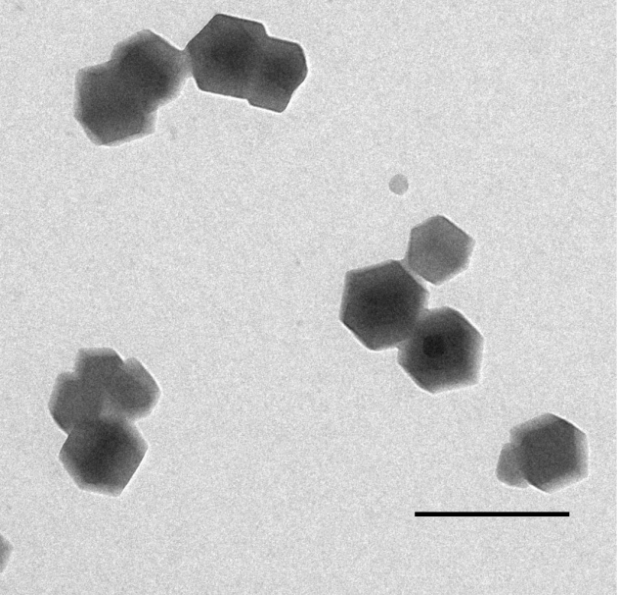

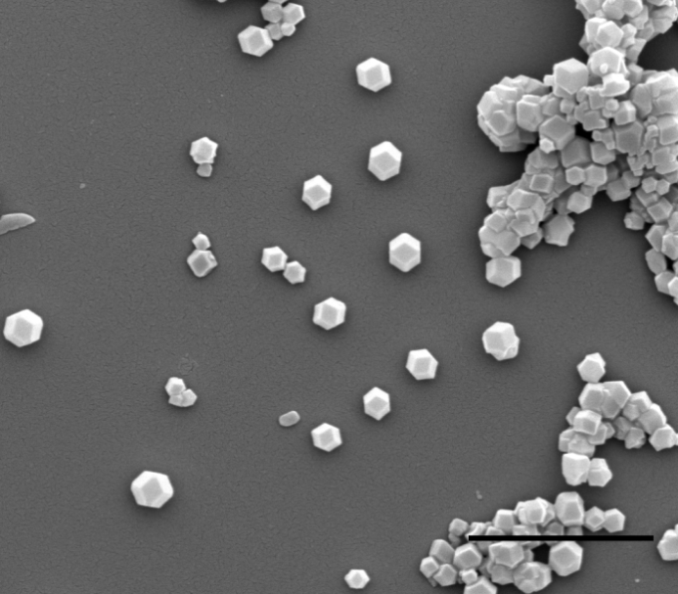
**

**b**

**(a)**

**(b)**

**2 µm**

**200 nm**

**Figure S3.** (a) TEM image of the ZIF-8 nano-crystals collected from the supernatant of the hybrid BCP@ZIF-8 solutions; (b) SEM image of the ZIF-8 NPs synthesized by mixing Zn(NO_3_)_2_ and 2-methylimidazole (HMeIM) in methanol at 70 ºC for 10 min.


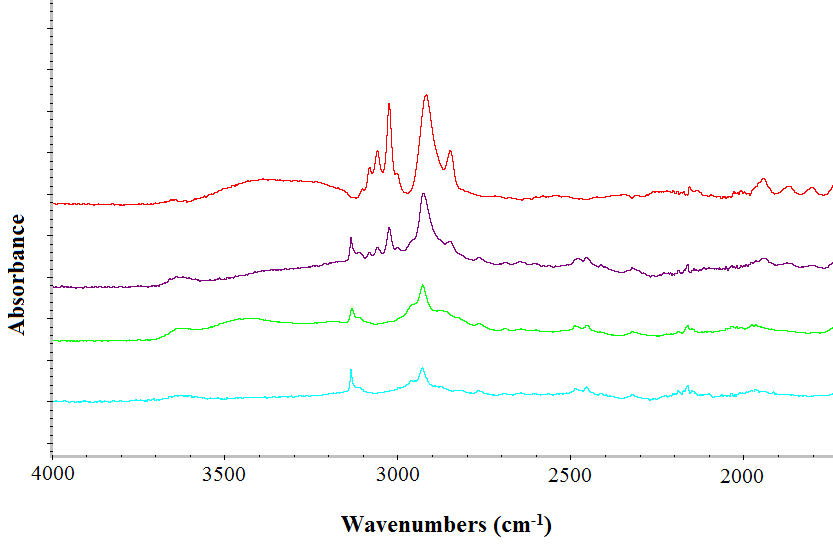


**BCP filament-shaped micelles**

**Hybrid BCP@ZIF-8 structures**

**Filamentous ZIF-8 NTs**

**ZIF-8 NPs nanoparticles**

**Figure S4.** FT-IR spectra of BCP filament-shaped micelles, hybrid BCP@ZIF-8 structures, filamentous ZIF-8 NTs and ZIF-8 NPs, respectively.


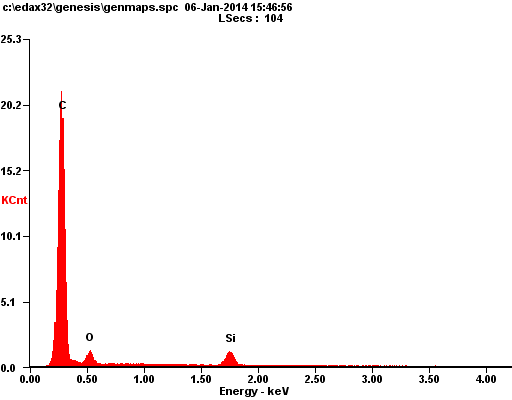


**BCP filament-shaped micelles**


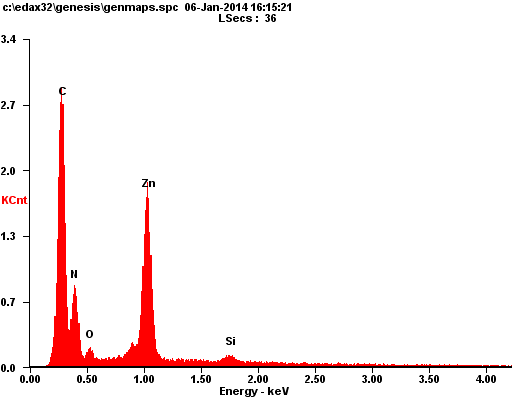


**Hybrid BCP@ZIF-8 structures**


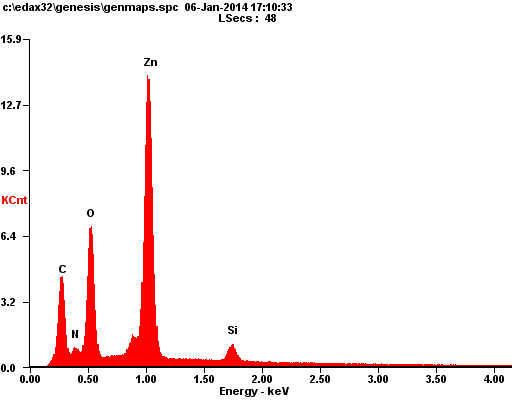


**Filamentous ZIF-8 NTs**

**Figure S5.** A series of EDX spectra of BCP filament-shaped micelles, hybrid BCP@ZIF-8 structures and filamentous ZIF-8 NTs.


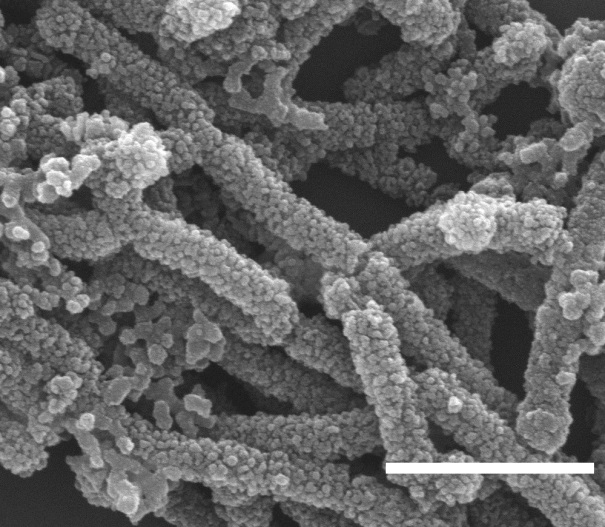


**1 µm**

**Figure S6.** SEM image of filamentous ZIF-8 NTs immersed in acidic phosphate buffer (pH = 5.0) at 37ºC for 30 min. All the surfaces of the nanotubes became loose and decomposed.


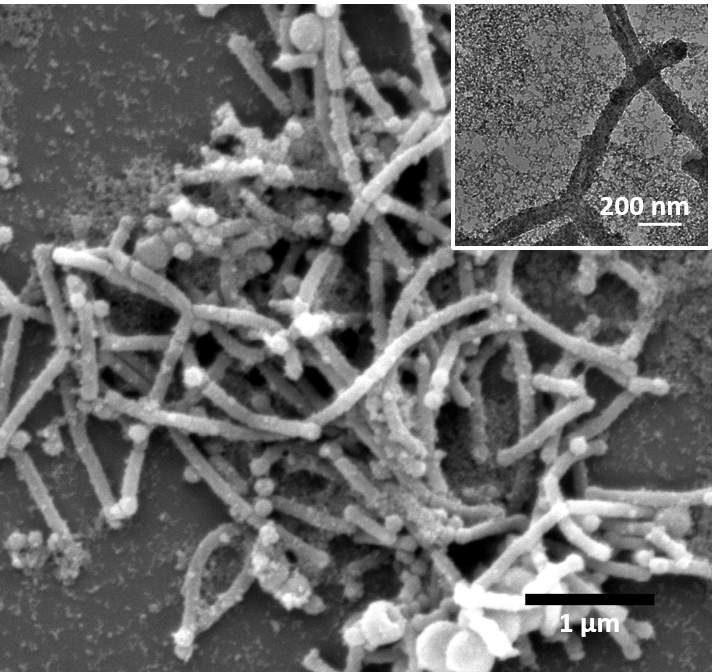


**Figure S7.** SEM image of filamentous ZIF-7 NTs. Inset is the TEM image of the ZIF-7 NTs which show the tubular characteristic.
